# Characterizing heterogeneous single-cell dose responses computationally and experimentally using threshold inhibition surfaces and dose-titration assays

**DOI:** 10.1038/s41540-024-00369-x

**Published:** 2024-04-18

**Authors:** Patrick C. Kinnunen, Brock A. Humphries, Gary D. Luker, Kathryn E. Luker, Jennifer J. Linderman

**Affiliations:** 1 Department of Chemical Engineering, University of Michigan, Ann Arbor, MI 48109 USA; 2 Department of Radiology, University of Michigan, Ann Arbor, MI 48109 USA; 3 Department of Biomedical Engineering, University of Michigan, Ann Arbor, MI 48109 USA; 4 Biointerfaces Institute, University of Michigan, Ann Arbor, MI 48109 USA

**Keywords:** Cancer, Pharmacology, Biochemical networks

## Abstract

Single cancer cells within a tumor exhibit variable levels of resistance to drugs, ultimately leading to treatment failures. While tumor heterogeneity is recognized as a major obstacle to cancer therapy, standard dose-response measurements for the potency of targeted kinase inhibitors aggregate populations of cells, obscuring intercellular variations in responses. In this work, we develop an analytical and experimental framework to quantify and model dose responses of individual cancer cells to drugs. We first explore the connection between population and single-cell dose responses using a computational model, revealing that multiple heterogeneous populations can yield nearly identical population dose responses. We demonstrate that a single-cell analysis method, which we term a threshold inhibition surface, can differentiate among these populations. To demonstrate the applicability of this method, we develop a dose-titration assay to measure dose responses in single cells. We apply this assay to breast cancer cells responding to phosphatidylinositol-3-kinase inhibition (PI3Ki), using clinically relevant PI3Kis on breast cancer cell lines expressing fluorescent biosensors for kinase activity. We demonstrate that MCF-7 breast cancer cells exhibit heterogeneous dose responses with some cells requiring over ten-fold higher concentrations than the population average to achieve inhibition. Our work reimagines dose-response relationships for cancer drugs in an emerging paradigm of single-cell tumor heterogeneity.

## Introduction

Despite numerous advances in cancer biology, target identification, and drug discovery and development, existing chemotherapy drugs generally fail to yield durable responses. Kinase inhibitors represent one promising new class of chemotherapeutic agents. These drugs are designed to inhibit the activity of an oncogenic kinase critical to transformation, proliferation, and/or survival. While kinase inhibitors have had some clinical success^1–3^, a variety of cell-intrinsic and cell-extrinsic resistance mechanisms have been identified, including variable drug distribution^4^, microenvironmental heterogeneity^5^, compensatory activation of other oncogenic signaling pathways^6–8^, and heterogeneity in the underlying population of cancer cells. Population heterogeneity can have multiple origins, including genetic and non-genetic differences among cells^9^. Non-genetic heterogeneity encompasses many aspects of cell behavior, including cell cycle state^10^, cell signaling pathways^11–13^, metabolism^14^, and migratory capacity^15,16^.

Several recent studies have examined the effects of heterogeneity in response to drugs that target specific signaling pathways. This work has mostly focused on the RAS/RAF/MEK/ERK pathway^17–21^, but others studied the response to inhibitors of CDK4/6^22^, the EGF receptor^23^, and Akt pathway inhibitors^24^. Cell barcoding and lineage tracing have revealed that prior to drug addition, single cells transiently exist in multiple subpopulations that display a spectrum of drug susceptibilities^20,21,25^. Upon treatment, the more drug-resistant subpopulations outgrow the drug-susceptible populations and may undergo large-scale changes in chromatin organization or genetic mutations, leading to durable drug resistance^25–27^. After surviving treatment, drug resistant cells continue to occupy a range of subpopulations depending on previous drug dose^28^ and may develop new vulnerabilities to specific treatments^29^. Notably, multiple approaches have shown that cells that continue to proliferate after MEK inhibition are able to maintain high ERK levels after drug exposure^17,20^, demonstrating a connection between persistent kinase signaling after targeted inhibition and survival. Hence, it appears that heterogeneity in kinase activity after targeted kinase inhibition is a crucial obstacle to cancer treatment.

Despite our growing understanding of single-cell drug responses, drug efficacy in cell-based assays is generally quantified using a population-scale dose-response experiment. In these experiments, cells are exposed to the drug of interest, and a relevant output (such as kinase activity) is measured^30^. Crucially, common measures of kinase signaling such as western blots involve lysing cells and aggregating protein levels from all cells into one sample, eliminating information about single-cell kinase activity. Drug dose is varied over several logs of concentration, and the output is plotted over the concentration range. After acquiring a dose-response curve, the data can be fit and parameterized using the following formula (Eq. 1) for a sigmoid:

1 $$R\left(d\right)={E}_{\max }+\left(\frac{{E}_{0}-{E}_{\max }\,}{1+{\left(\frac{d}{E{C}_{50}}\right)}^{{HS}}}\right)$$

where *R* is the response, *d* is the drug concentration, *E*_max_ is the response at infinite concentration, *E*_0_ is the initial response, EC_50_ is the concentration required to achieve 50% of the maximal response, and Hill slope (HS) is a measure of the steepness of the sigmoid^31^. Using these parameters, dose responses can be rapidly compared among drugs, cell lines, and conditions. Typically, decreasing the EC_50_ or increasing the *E*_max_ is used as evidence of increased drug potency^32–34^. More recent work has improved dose-response analysis by correcting for differences in cell growth rates and drug response times^35,36^. However, these approaches reveal very little about the drug response heterogeneity present in a population of cells^37^. Drug-cell line combinations with high *E*_max_ have been associated with the presence of resistant subpopulations^37^, but dose–response curves rely on measurements that are fundamentally population level.

One target of novel kinase inhibitors is phosphatidylinositol-3-kinase (PI3K), which activates downstream effectors including Akt and mammalian target of rapamycin complex 1 (mTORC1)^38^. The PI3K/Akt/mTORC1 pathway controls cell proliferation in response to growth factor signaling. PI3K activity is opposed by the Phosphatase and Tensin Homolog (PTEN) phosphatase. Together, mutations that either constitutively activate PI3K or inactivate PTEN are present in 40% of breast cancer cases, occurring more commonly in estrogen receptor (ER) positive cancers^39^. Based on the prevalence of PI3K activation in ER+ breast cancer, specific inhibition of PI3K is a promising therapeutic strategy. More recent PI3K inhibitors (PI3Kis) have emerged that target specific PI3K isoforms, such as alpelisib, which targets PI3Kα. Patterns of PI3K isoform expression are cell-specific, but PI3Kα is commonly mutated or overexpressed in breast cancer and many other malignancies^40^. Other drugs, including omipalisib, simultaneously inhibit PI3K and mTOR, which shares a similarly shaped kinase domain with PI3K^41^. In 2022, alpelisib became the first clinically approved PI3K inhibitor for metastatic ER+ breast cancer^2^. Though effective in prolonging survival, alpelisib does not cure metastatic disease. Treatment failures can occur due to the activation of compensatory signaling pathways in cancer cells^42,43^. Patients also may discontinue treatment because of severe side effects, such as systemic hyperglycemia due to interruption of insulin signaling and glucose uptake in tissues^44^. However, unlike BRAF V600E mutations and inhibition of the MAPK pathway, less is known about how a heterogeneous overall population of breast cancer cells responds to PI3K inhibition.

To better understand the currently insurmountable challenge of tumor heterogeneity and drug resistance, we analyzed single-cell drug responses by measuring dose-titration curves in individual cells. We first developed a computational model to compare how different distributions of dose-response parameters in a population would affect the population-level dose response. Our model reveals a major shortcoming in standard dose–response assays: markedly different population distributions can generate indistinguishable dose responses. Inspired by this shortcoming, we introduce an approach that is sensitive to differences in the underlying population distributions. This analysis, which we term threshold inhibition curves, can be performed on any single-cell dose response dataset. To demonstrate the utility of the approach experimentally, we combined fluorescent single cell kinase reporters with an experimental design in which breast cancer cells are exposed to increasing doses of PI3K inhibitors alpelisib or omipalisib. We acquired single-cell measurements of Akt and ERK signaling pathway activities in multiple breast cancer cell lines at a wide range of PI3Ki doses on time-scales from minutes to h. Our measurements revealed that MCF-7 cells respond rapidly to PI3Ki, while Vari-068 cells do not. We were able to extract single-cell dose-response curves from MCF-7 cells and fit dose-response parameters to individual cell drug responses. Our approach revealed remarkable heterogeneity in dose responses to clinically relevant PI3K inhibitors. Surprisingly, we observed that a substantial subpopulation (~10%) of ER+ MCF-7 breast cancer cells exhibit EC_50_ values more than 10 times greater than the population EC_50_ for omipalisib. Our work demonstrates that ubiquitous population-scale measurements of drug response obscure subsets of drug-resistant cancer cells that may drive recurrent breast cancer.

## Results

### Heterogeneous single-cell dose responses cannot be differentiated by population measurements

**Fig. 1:**
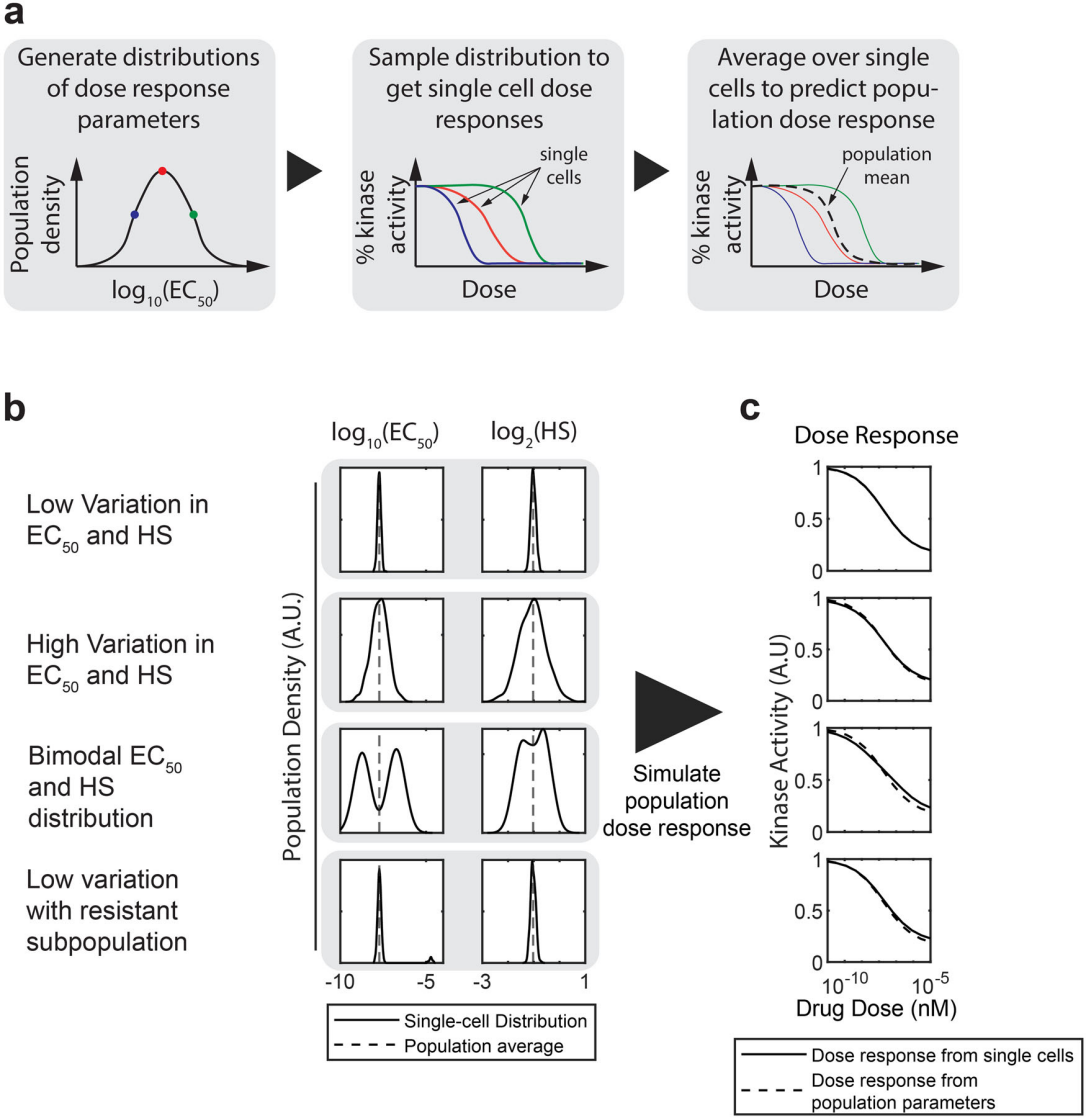
Modeling dose responses from heterogeneous populations. **a** Schematic of model showing how we generate individual dose responses by sampling from distributions of parameters and then averaging across sampled cells to simulate the population dose response. **b** Simulated population densities (smoothed histograms of the proportion of cells with a given dose response parameter value) of EC_50_ and Hill slope parameters for four different hypothetical populations. Dashed lines represent the EC_50_ or Hill slope inferred from population level data, while solid lines are the simulated distribution. Note that all four populations have almost identical population means. **c** The resulting overall dose responses from averaging over cells sampled from each population in (**b**). Solid lines represent the average from sampling each population 1000 times, while the dashed line represents the dose response from the measured population dose response parameters (dashed lines in **b**).

We began with a modeling approach to analyze what population-level dose response experiments might reveal about the underlying heterogeneous cellular populations. Since population-level dose response measurements are common, understanding their ability to detect single cell heterogeneity is vital to their interpretation and use. We hypothesized that many different distributions of dose responses, which could include resistant outlier populations or other types of variation, could yield indistinguishable population-level dose responses. To test this hypothesis, we built a simple computational model to compare single-cell and population-level dose responses. We assumed that a kinase inhibitor could cause a continuous change in the level of active kinase in a cell, and each cell in a population could have its own dose response curve, parameterized by an EC_50_, Hill slope, *E*_max_, and *E*_0_. From these assumptions, we inferred that a population-level dose response would result from averaging the amount of active kinase across all cells in a population. To construct a model based on these assumptions, we first simulated distributions of dose response parameters, then sampled these distributions to get single-cell dose response parameters (Fig. 1a). We put each set of single-cell dose response parameters into the dose response equation (Eq. 1) to generate single-cell dose responses and then averaged over the entire population of cells to get the simulated population dose response.

For our simulation, we started with dose response parameters (EC_50_, Hill slope, *E*_max_, and *E*_0_) for MCF-7 cells exposed to omipalisib gathered from previous experiments^32,37^. Using those population-level parameters as the mean value, we generated distributions for EC_50_ and Hill slope. We generated four different distributions, corresponding to populations with low and high variability in EC_50_ and Hill slope, a population with a bimodal distribution of EC_50_ values, and a population with a small subpopulation of “resistant” cells that had a much higher EC_50_ and *E*_max_ than the bulk population (Fig. 1b). We sampled each set of distributions 1000 times, corresponding to 1000 individual cells, and averaged across the simulated population to generate a simulated population dose response. Our simulation shows that the population-level dose responses we simulate approximate the mean behavior of the population. However, the population-level dose response is essentially identical across populations, independent of the underlying distributions present in the population (Fig. 1c). This result is intuitive but demonstrates that if cell-to-cell variability is present in drug response, population-level measurements of drug response cannot capture or characterize this variability.

Using our simulation, we next asked to what extent the population-level dose response represents the behavior of any individual cell in the population. This question was prompted by observations from developmental biology and toxicology that a population of binary responders (i.e., high Hill slope behavior) with different thresholds (EC_50_ values) will lead to a gradual population response (i.e., low Hill slope)^45,46^. Using the same model, we generated distributions of cells with a unimodal distribution of EC_50_ and Hill slope values with the Hill slope distribution centered at relatively steep Hill slope values (mean Hill slope = 3.16, log_2_(mean Hill slope) = 1.66). We sampled these distributions and generated a simulated population dose response (Supplementary Fig. 1a), and then fit Eq. 1 to the simulated population dose response. The distribution of EC_50_ and Hill slope values sampled and a vertical line representing the EC_50_ and Hill slope acquired from the population fit are shown in Supplementary Fig. 1b and Supplementary Fig. 1c. Comparing the population fit (blue vertical line) and the underlying distribution (red curve) reveals that the Hill slope inferred from the population represents less than 5% of cells in the population, and most cells have a much higher Hill slope. A steep Hill slope can reflect strong feedback loops within the cell, and systemically underestimating the actual Hill slope of response present in single cells could lead to misinterpretation of population-level data. More broadly, our simulation suggests that population-level dose responses obscure underlying heterogeneity and may not actually correspond to any single-cell behavior present in the population.

### Threshold inhibition curves and surfaces characterize heterogeneous dose responses

Given that population-level dose response curves cannot capture heterogeneity in populations, we propose a new way of characterizing dose-dependent kinase inhibition based on single-cell measurements: *threshold inhibition curves*. Instead of plotting population-averaged kinase activity as a function of dose, we instead define a threshold of kinase inhibition and for each dose plot the proportion of cells that fall below the defined threshold.

**Fig. 2:**
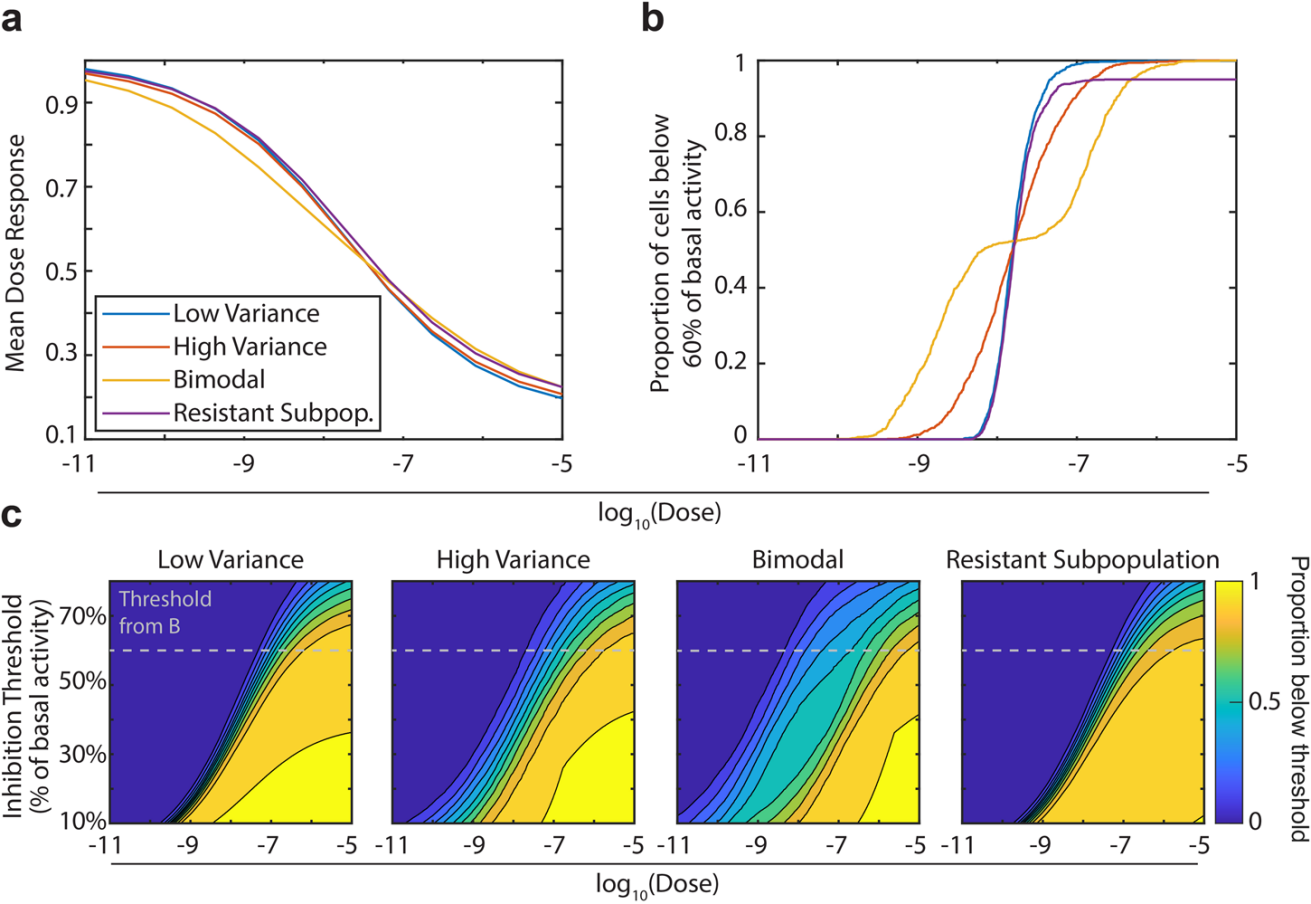
Threshold inhibition curves and surfaces capture heterogeneity in dose response. **a** Population averaged dose responses from simulated populations in Fig. 1. **b** Threshold inhibition curve for the same populations in Fig. 1. The curves were calculated by sampling each population 1000 times, and then calculating the proportion of the population inhibited to below 60% of their basal signaling at each dose. **c** Threshold inhibition surfaces, calculated as in (**b**) but with a varying threshold. A dashed line has been added at the 60% threshold, profiles along these lines correspond to the lines in (**b**).

We first deploy this analysis on the hypothetical populations shown in Fig. 1. We once again sampled each population 1000 times to generate 1000 simulated single-cell dose–response curves. Then, we defined a threshold, such as 60% of the cell’s basal activity, and calculated the proportion of cells with signaling below that level at a given dose. Directly comparing traditional dose responses with threshold inhibition curves demonstrates their utility (Fig. 2a, b). As observed previously, the population-averaged dose response curves are practically indistinguishable for the four populations described by different parameter distributions in Fig. 1 (Fig. 2a). However, by examining threshold inhibition curves, the four populations are readily distinguished (Fig. 2b). The low and high variance populations reach 50% of cells inhibited at the same dose, but the higher variance population has cells that reach that threshold at lower doses, and cells that remain uninhibited until higher doses. Similarly, the two underlying populations within the bimodal population are clearly distinguished. Finally, the resistant subpopulation can be identified because the curve does not reach 100% of cells inhibited at any dose in the simulation.

It is unclear what degree of inhibition is required to induce a desired phenotype in vitro or in vivo, and the 60% threshold in Fig. 2b was chosen as an illustrative example. Thus, we expanded our analysis by varying the threshold required for inhibition of cells, creating a *threshold inhibition surface* dependent on both the selected threshold and the applied dose (Fig. 2c). This further reveals different characteristics of the populations. Compared to the high variance population, the low variance population has less heterogeneity at all thresholds. For the low-variance population, cells are inhibited at a wider range of doses at higher inhibition thresholds. Similarly, the bimodal populations are only apparent at inhibition thresholds between 30% and 50%, while at very high or very low thresholds the population appears more unimodal. The threshold inhibition surfaces again show that the population with a resistant subpopulation is not fully inhibited even at high doses.

### Dose titration assay enables the measurement of single-cell dose responses

After demonstrating computationally that population dose response measurements can mask single-cell heterogeneity, we next tested the ability to quantify the heterogeneity that cells show in response to a clinically relevant drug. We measured single-cell dose responses to PI3Kis alpelisib and omipalisib. We tested these PI3Kis on MCF-7 and Vari-068 breast cancer cell lines. MCF-7 cells are an ER+ cell line with constitutively active PI3K pathway signaling due to a mutation in the catalytic subunit of PI3K. Vari-068 cells have constitutive PI3K pathway activity due to a mutation in PTEN^13^. We used kinase translocation reporters (KTRs) for kinases Akt and ERK to measure single-cell signaling activities^13,47–49^. KTRs rapidly and reversibly translocate between the cytoplasm and nucleus in response to the activity of a specific kinase. KTRs are quantified by the logarithm of the ratio of fluorescent intensity between the cytoplasm and nucleus (log_2_(CNR)), with higher values indicating greater signaling activity. Akt is directly downstream of PI3K and is a commonly measured output for PI3K inhibition. ERK is less directly associated with PI3K, but it is another commonly activated kinase in cancer and an important therapeutic target. Furthermore, we and others have found substantial crosstalk between the ERK and Akt pathways, and there is evidence that PI3K inhibition can also inhibit ERK pathway activity^13,49,50^.

To quantify how MCF-7 and Vari-068 KTR cells respond to individual PI3Ki concentrations, we stably expressed Akt and ERK KTRs in both cell lines along with a stable histone-2B nuclear marker. We first exposed cells to a range of individual concentrations of omipalisib or alpelisib in live-cell microscopy experiments. Using automated image processing, we extracted single-cell signaling trajectories for ERK and Akt. In untreated conditions, the ratio of cytoplasmic to nuclear fluorescence intensity is higher in the Akt channel than the ERK channel (Supplementary Fig. 2a, Supplementary Fig. 3a), which suggests greater activation of Akt by constitutively active PI3K signaling in these cells. Averaging among all cells in the population revealed dose-dependent Akt and ERK inhibition (Supplementary Fig. 2b, Supplementary Fig. 3b). Omipalisib was more potent than alpelisib (achieving measurable inhibition at lower doses) in both cell lines, consistent with previous measurements^32^. We observed differences in deactivation kinetics between MCF-7 and Vari-068 cells. After inhibition, MCF-7 cells reached steady inhibition of both Akt and ERK within 60 min (Supplementary Fig. 2b). However, Vari-068 cells responded more slowly, reaching full inhibition to high inhibitor doses after 2–3 h (Supplementary Fig. 3b). Differences between cell lines may be secondary to distinct mutations that constitutively activate the PI3K pathway. These results suggest that our MCF-7 and Vari-068 KTR cells detect continuous changes in kinase signaling in response to PI3K inhibition.

**Fig. 3:**
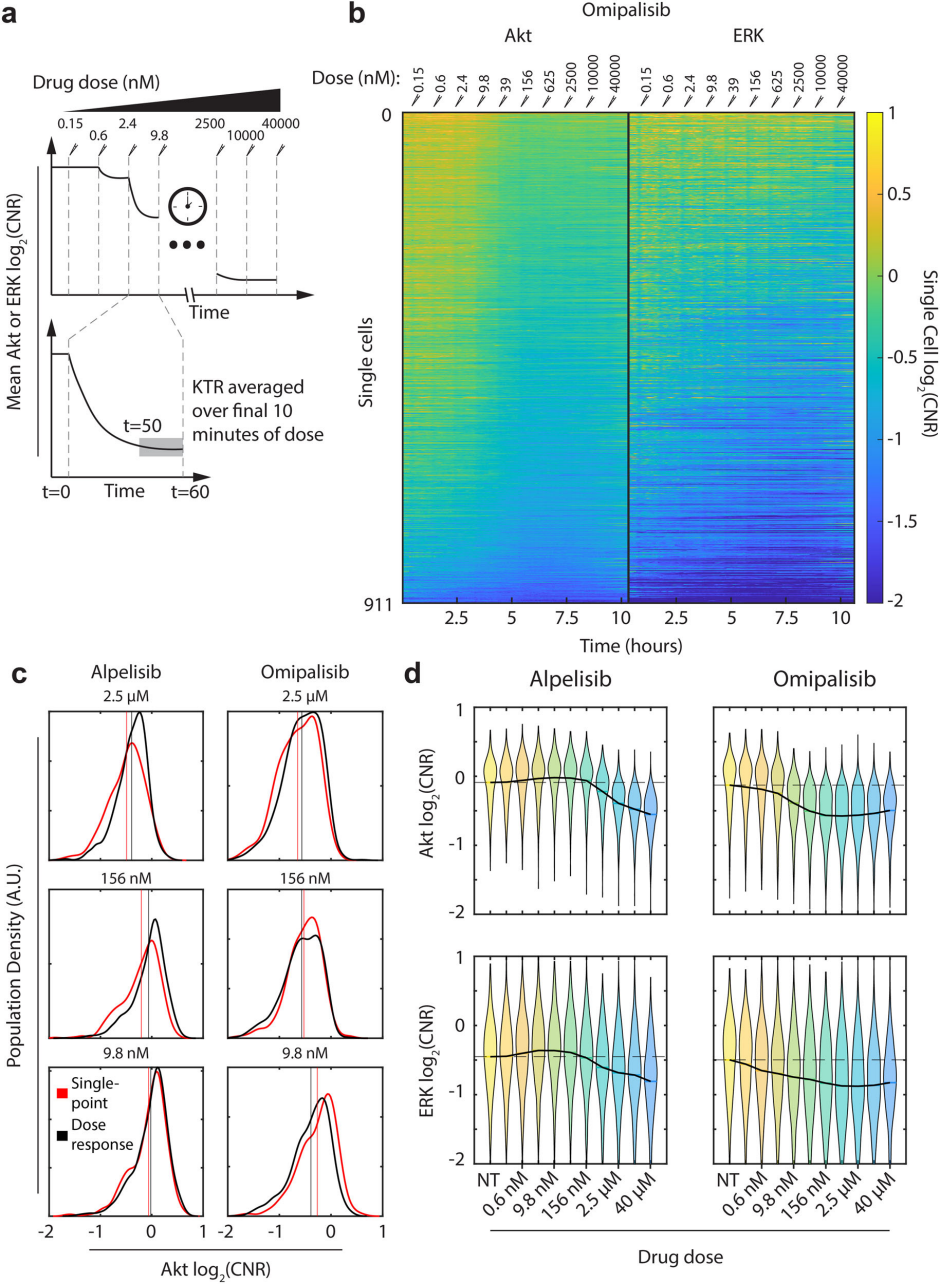
Measuring dose responses in individual cells. **a** Experimental approach. Cells are exposed to a single concentration of alpelisib or omipalisib for an h, and the single-cell response is averaged over the last 10 min of exposure. Then, a higher concentration is added. Each concentration is 4× greater than the previous one. Concentrations range from 150 pM to 40 μM. **b** Kymograph showing single-cell trajectories of each MCF-7 cell measured in an omipalisib dose-response experiment. Each row is a single cell, and each column is a different time point, with color representing the signaling activity. Paired Akt and ERK trajectories are shown, with Akt on the left and ERK on the right. Cells are sorted by the area under the curve of their Akt trajectory. The dose of omipalisib at each timepoint is shown at the top of the kymograph. **c** Comparison of Akt signaling distributions when exposed to a given drug concentration in a single-point experiment (red) and a dose response experiment (gray). Each distribution was generated using kernel smoothing. Vertical lines indicate the population mean of each distribution. **d** Akt (top) and ERK (bottom) dose-dependent signaling activity in response to alpelisib (left) and omipalisib (right). In each plot, the distribution of signaling activity is shown as a violin, while the mean dose response is indicated with a solid black line. A horizontal dashed black line is included at the untreated mean to guide the eye. The full list of doses used in the figure, from left to right in increasing order, is: No treatment (NT), 0.15 nM, 0.6 nM, 2.4 nM, 9.8 nM, 39 nM, 156 nM, 625 nM, 2.5 μM, 10 μM, 40 μM.

Exposing cells to a single dose of PI3Ki reveals individual cell signaling levels, but it does not reveal dose response parameters. We would like to understand the distribution of dose response parameters within a population so that we can detect the presence of outlier cells requiring high concentrations of drug to be inhibited. Thus, we next measured dose responses in single cells by exposing the same population of cells to increasing doses of each PI3Ki, which we refer to as a dose titration. For each dose, we imaged cells continuously for 1 h. Since MCF-7 cells reach a new steady state rapidly after exposure to a new dose, we focus our analysis on them. Our experimental approach is demonstrated in Fig. 3a. We used our automated image processing pipeline^13,49^ to extract values for Akt and ERK kinase activities from individual cells tracked for the duration of the experiment.

We next investigated how cells varied in dose-dependent deactivation of Akt and ERK. We examined the trajectories of each individual cell exposed to increasing doses of omipalisib using a kymograph to plot the time-domain signaling activity of each cell (Fig. 3b). Since we added increasing doses of omipalisib throughout the experiment, we were also able to associate specific timepoints with specific drug doses, which are shown at the top of the kymograph. We sorted cells by the area under the curve of their Akt trajectory, so cells with higher overall Akt are at the top of the kymograph. Our data show substantial heterogeneity in basal state and inhibition response. Most cells show substantial Akt inhibition at an omipalisib dose of 9.8 nM, but many respond well before that dose. The degree of Akt inhibition varies from cell to cell. ERK also shows heterogeneity in responses with some cells almost unaffected by omipalisib and others demonstrating substantial inhibition.

### Dose-titration Akt inhibition matches single-point experiments

Our goal is to measure how individual cells will respond to any dose of PI3Ki. Thus, it is vital to ensure that our drug titration experiments correspond to single-dose experiments. Specifically, we wondered if dose-titration experiments might provoke compensatory signaling limiting later inhibition, or if spreading doses over time could enhance their effects. We exposed MCF-7 cells to three different single doses of omipalisib or alpelisib and extracted the distribution of Akt log_2_(CNR) values 1 h after PI3Ki addition. We compared these distributions with the equivalent time-point in dose response experiments (1 h after the corresponding dose was given). We find that the distribution of Akt signaling activity in dose-titration experiments appears to match single-point distributions, with no systematic increase or decrease in inhibition. These results suggest that for MCF-7 cells exposed to alpelisib or omipalisib, the levels of inhibition observed in dose-titration experiments do correspond to more traditional single-point measurements. Based on this observation, we can plot the data in Fig. 3b as dose-dependent, instead of time-dependent, signaling. Figure 3d summarizes our results using violin plots to demonstrate the distribution of signaling activity at each dose, along with the mean dose response (black line). Our ability to extract dose-dependent Akt and ERK signaling from the same cells enables us to measure full dose-response curves in single cells.

We performed identical experiments in Vari-068 cells, applying increased doses of alpelisib or omipalisib every h for ten total doses. However, as previously observed (Supplementary Fig. 1), we found slower deactivation of Akt in response to omipalisib in Vari-068 cells. Akt deactivation in response to alpelisib was only observed at very high concentrations. Due to the delayed kinetics of Akt deactivation, a 1-h increment between doses was not enough time for Vari-068 cells to reach a new steady state, and there was a larger discrepancy between single-point and dose-response signaling distributions than in MCF-7 cells (Supplementary Fig. 4 for Vari-068 cells, compared to Fig. 3c for MCF-7 cells). We also compared the distribution of Akt signaling in Vari-068 and MCF-7 cells exposed to high doses of alpelisib and omipalisib (highest doses from Supplementary Fig. 2 and Supplementary Fig. 3). We found that there were similar levels of heterogeneity of Akt and ERK activities in both cell lines after full inhibition, demonstrating that heterogeneity in both basal state and in response to PI3Ki is a feature of both cell lines (Supplementary Fig. 5).

### Measuring dose-response parameters in single cells

**Fig. 4:**
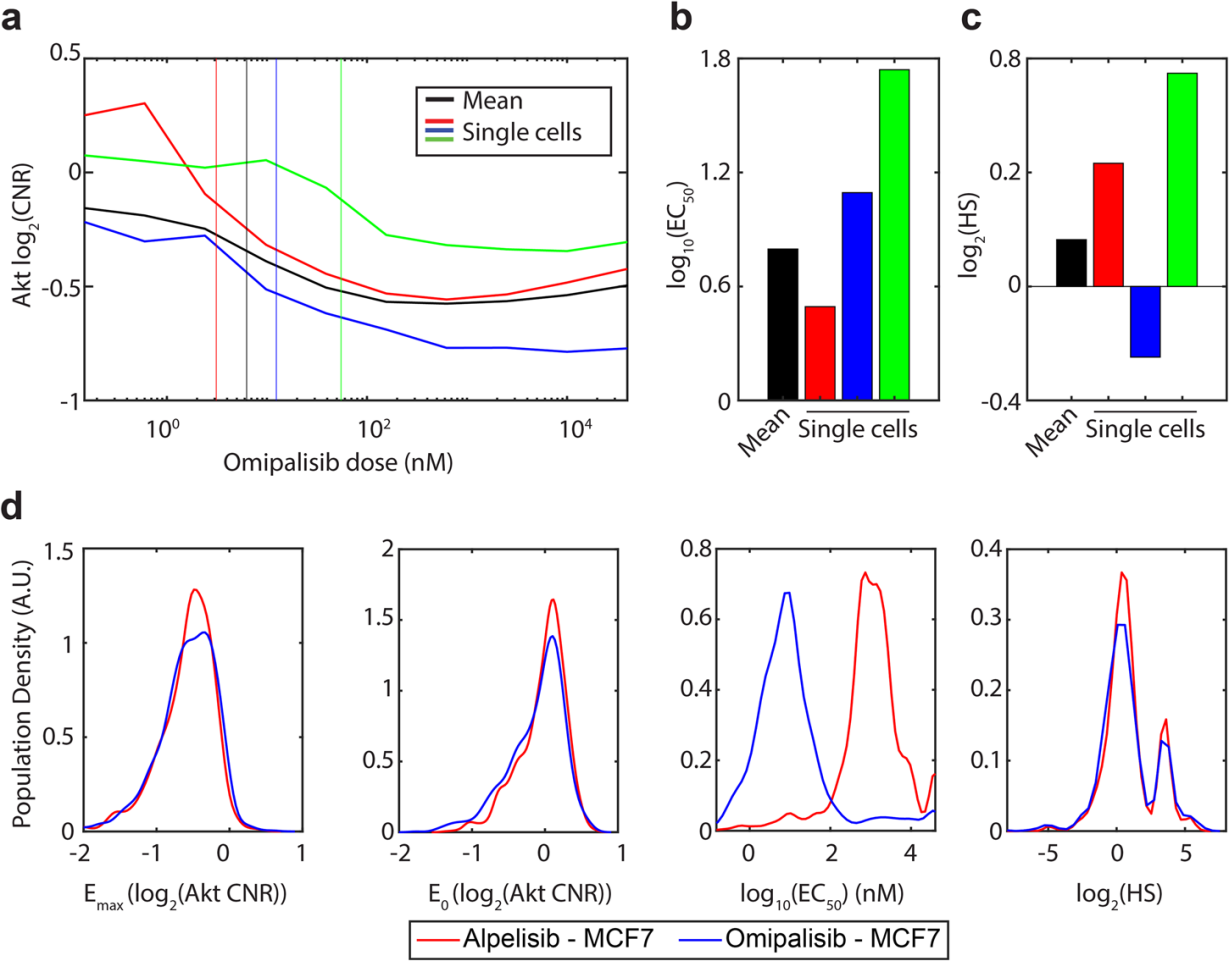
Characterizing heterogeneous dose responses. **a** Mean dose response (from Fig. 1d, top right) and three representative individual dose responses for MCF-7 cells exposed to Omipalisib. Vertical lines indicate the EC_50_, obtained by fitting Eq. 1 to each individual dose response. **b**, **c** Bar graphs comparing the EC_50_ and Hill slope parameters for the mean and single-cell dose responses shown in (**a**). **d** Distributions of dose response parameters extracted from single cells for both alpelisib and omipalisib. Distributions are the kernel-smoothed density of the underlying data. For alpelisib *N* = 1040 cells and for omipalisib *N* = 911 cells.

We next characterized dose responses in single MCF-7 cells. We fit the dose-response equation (Eq. 1) to each cell in the population exposed to either alpelisib (*N* = 1040 cells) or omipalisib (*N* = 911 cells). Examining individual cells reveals important deviations from the population-averaged behavior (Fig. 4a). Individual cells can have EC_50_ values at least ten times greater than the population average (Fig. 4b), indicating that inhibition requires higher drug concentrations. The population average hill slope is slightly greater than 1 (log_2_(Hill Slope) more than 0), showing relatively steep transitions from activation to inhibition, but some cells have more shallow transitions (Fig. 4c). At the single-cell level, this could relate to the strength of feedback loops and compensatory pathways connecting PI3K inhibition with downstream Akt activation. We further quantify this observation by plotting the single-cell dose-response parameters distribution in Fig. 4d. For both drugs, the E_0_ distributions are identical, reflecting that no drug has been applied at this point. Both drugs can achieve the same amount of Akt inhibition at high doses. The distribution of observed EC_50_ values varies substantially between drugs, with omipalisib exhibiting approximately 100-fold greater potency than alpelisib, matching the population-aggregated measurements (Fig. 4d). Notably, there is substantial variation in EC_50_ within each population. Though most cells show an EC_50_ for omipalisib around 10 nM, 99 of the observed 911 cells (11%) exhibit an EC_50_ greater than 100 nM. This observation suggests that dosing regimens based on achieving a tissue concentration based on population-level dose responses may not inhibit a large portion of cancer cells. Furthermore, although omipalisib is clearly more potent than alpelisib based on previous measurements^32^ and population-averaged dose responses measured here, single-cell distributions of potency overlap significantly, complicating the comparison.

### Single-point measurements do not predict dose-response measurements

**Fig. 5:**
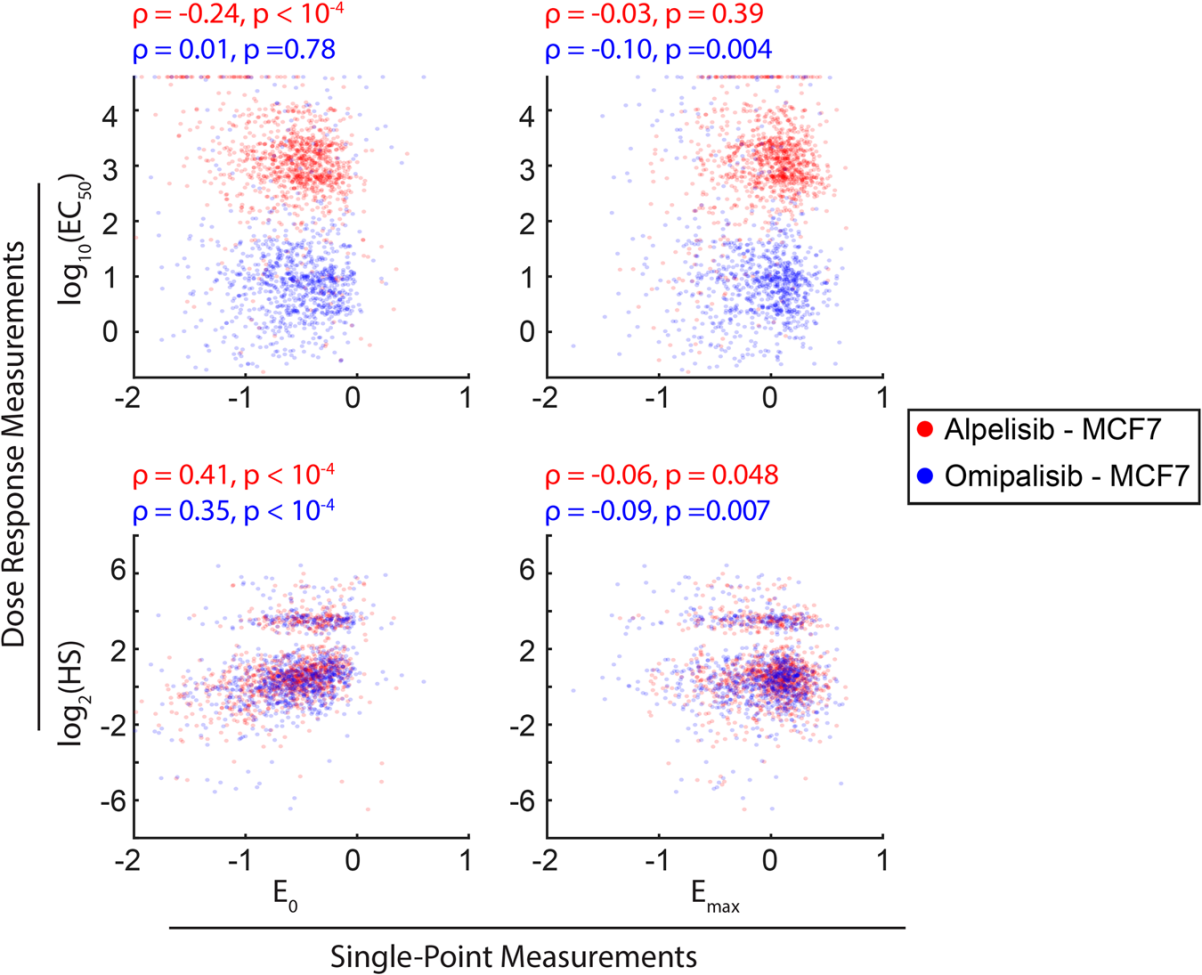
Single-point measurements do not predict dose-response parameters. Correlation between single-point parameters (*E*_0_, *E*_max_) and dose-response parameters (Hill slope, EC_50_) for MCF-7 cells exposed to an omipalisib dose-titration of either alpelisib (red) or omipalisib (blue).

After observing substantial variability in Akt dose responses, we next sought to quantify to what extent and how different dose response parameters were correlated for MCF-7 cells exposed to alpelisib or omipalisib. We consider *E*_0_ and *E*_max_ to be “single-point” measurements, since they can be measured using a variety of single-cell modalities by simply exposing cells to either very low or very high drug concentrations. Meanwhile, measuring EC_50_ or Hill slope requires measurements of the same cell across a range of concentrations, necessitating more complex live-cell methods. For both alpelisib and omipalisib, there was very weak or no correlation between either of the single-point measurements and EC_50_ or Hill slope (Fig. 5). Spearman correlation coefficients were generally weak and nonsignificant, except for a weak correlation between E_0_ and log_2_(Hill Slope). This further supports that our dose-titration assay produces previously unobserved measurements about single-cell drug effects.

### Threshold inhibition surfaces can characterize heterogeneous dose responses in experimental populations

**Fig. 6:**
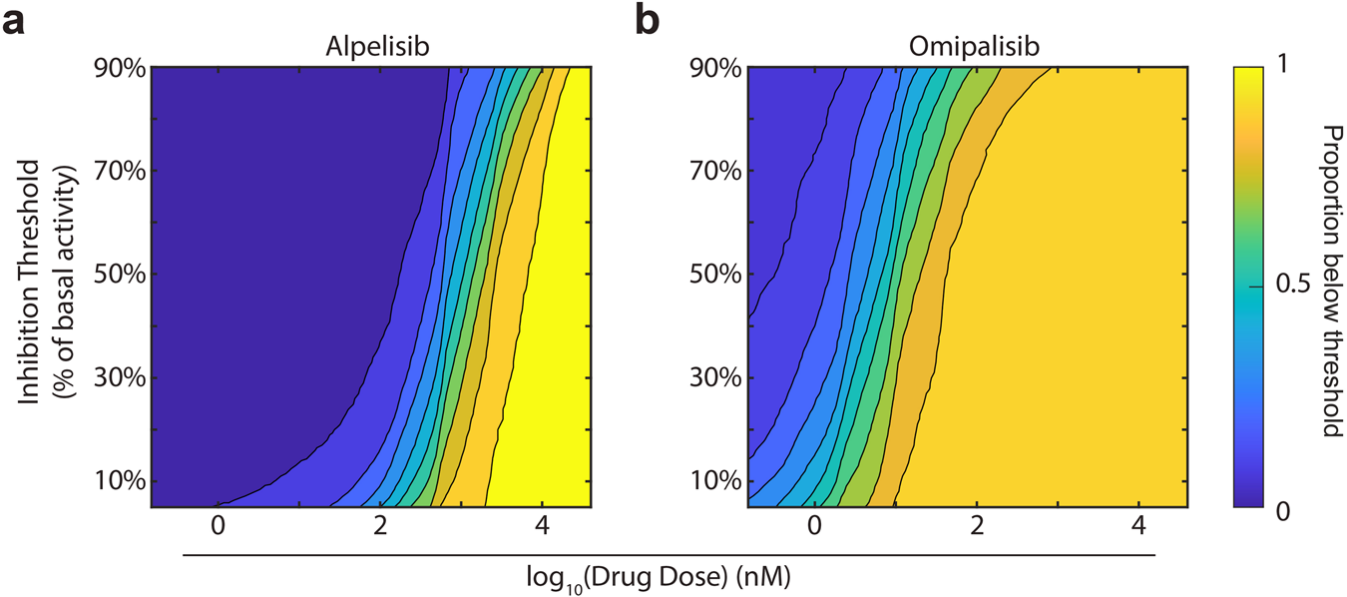
Threshold inhibition surfaces for experimental populations. We calculated threshold inhibition surfaces as in Fig. 2 for experimental populations of MCF-7 cells exposed to alpelisib (**a**) or omipalisib (**b**). In (**a**), *N* = 1040 cells. In (**b**), *N* = 911 cells.

Threshold inhibition surfaces (Fig. 2c) are based on applying thresholds to dose-response curves from individual cells. Thus, we were able to calculate threshold inhibition surfaces for our experimentally measured populations of MCF-7 cells exposed to alpelisib or omipalisib (Fig. 6). These response surfaces fully characterize the heterogeneity observed in our experiments. Comparing alpelisib and omipalisib, we again see that for any threshold inhibition, omipalisib is substantially more potent. In both populations, the threshold affects the observed heterogeneity, as illustrated by the difference between the concentrations where cells are initially inhibited and fully inhibited. For alpelisib, at low thresholds (around 30%) there is a substantially wider range of doses which achieve some degree of inhibition than at higher thresholds. Furthermore, we expect that omipalisib will have a more heterogeneous response since the range of doses causing inhibition is wider than alpelsib. We can also use these surfaces to interpret how increasing dose will affect heterogeneous populations by examining the slope of the contour lines. The linear portion of the contour lines for both alpelisib and omipalisib demonstrate that to inhibit another 10% of cells in a population, ~1.1x more drug is needed. Taken together, this work provides a proof of concept for using threshold inhibition surfaces to characterize and visualize the heterogeneity in dose response to multiple drugs.

## Discussion

Heterogeneous responses of individual tumor cells are a major obstacle to cancer therapy. Our work adds new tools to uncover this heterogeneity, and provides a proof-of-concept example using MCF-7 breast cancer cells and their responses to two clinically relevant PI3Kis. First, we present a simple model that enables us to explore the correspondence between population dose responses and the dose responses of the individual cells making up this population. We also present a method to characterize and visualize heterogeneous dose responses, which we term dose response surfaces. Dose response surfaces provide a quantitative measure of the proportion of cells that will be inhibited given a drug dose and a specific level of inhibition required. Dose response surfaces can be created quickly using dose-response data gathered from individual cells (experimental or simulated) and can be used to compare the level of overall inhibition (i.e., potency) and heterogeneity between multiple drugs. We envision dose response surfaces as being particularly useful when combined with experimental^51^ or simulated^52^ data of spatiotemporal drug distributions inside a tumor. These data show that cells experience substantial variations in drug concentrations over space and time in a tumor. We expect that heterogeneity in single-cell dose responses would exacerbate this heterogeneity and increase the likelihood that some cells exposed to low or transient drug doses survive. Our results also show that drug doses can be raised to inhibit greater than 90% of cells. However, these doses would likely demonstrate intolerable toxicity.

Second, we present a proof-of-concept example for experimentally measuring single-cell dose responses. While single cell measurements can be acquired after a single drug dose using a variety of methods, such data reveal little about drug effects since the pre-inhibition state is unknown. It can also be unclear whether heterogeneity measured in this way is due to stochastic noise or pre-existing cell state^53^. Using our dose-titration assay to acquire measurements of single cells at multiple doses, we demonstrate that single MCF-7 cells have markedly different dose responses to the PI3Kis alpelisib and omipalisib. This is similar to findings in previous studies in which cells were exposed to escalating doses of a biological stimuli (acetylcholine or insulin growth factor) and a downstream readout (calcium or Akt signaling) was measured^54,55^. In both cases, individual cells exhibited consistent variation from the mean, as opposed to random variation to each stimulus. We also identify systemic, rather than random, differences in cellular responses to kinase inhibition.

The source of signaling heterogeneity is not fully understood. We and others have found that signaling heterogeneity can be explained by differences in pre-existing state, utilizing two fundamentally different approaches. One approach is to use high-dimensional endpoint measurements, including single cell sequencing or high-content imaging, to characterize the variability present in cellular populations exposed to different inputs^56,57^. This approach yields high-dimensional landscapes of cellular heterogeneity and has been used to predict single-cell dose responses to epidermal growth factor^56^. However, endpoint measurements require killing cells, making it impossible to associate a high-dimensional characterization of cell state with later behavioral measurements. Another approach, employed by us and others, is to use live-cell imaging to measure signaling dynamics in individual cells and fit kinetic models of signaling to individual cells, thus inferring cell state from the model parameters that best fit each cell^12,49,58,59^. This approach is amenable to dynamic data, and cell states can be inferred and associated with later behaviors. However, inferring cell states requires dynamic models as opposed to direct measurements. Both approaches predict that, due to differences in cell state, cells should have different intrinsic sensitivity to kinase inhibition. Our results validate these predictions. Furthermore, by comparing how different preconditions affect distributions of measured dose responses, the assay presented here could help unify the two approaches to identifying cell states by connecting cell state measurements to downstream behaviors.

Another outstanding challenge in understanding resistance to targeted therapy lies in unifying the timescales of various observations. Previous work using live cell microscopy has revealed a repeatable series of events occurring over several h to days, including specific transcriptional changes, the induction of stress signaling pathways, and atypical cell replication, which enable persistent cells to continue growing^17,18^. Lineage tracking enables resistant clones to be tracked over several days^20^. Finally, França et al. tolerized cells to increasing drug doses over several weeks to months, revealing a diverse set of transcriptional changes in resistant cells that are dependent on the tolerizing dose^28^. Meanwhile, our assay reveals heterogeneity in cellular response to drugs over minutes to h. However, we do not track cells over longer time periods or determine which cells re-enter the cell cycle. Based on previous reports of compensatory signaling activation^43^, we speculate that at least some cells with a low EC_50_ (meaning they are easily inhibited) would reactivate signaling. Thus, future work should focus on comparing three populations of cells: (1) Those which initially respond to the inhibitor and remain inhibited; (2) those which respond and reactivate kinase signaling due to compensatory signaling mechanisms; and (3) those which do not respond to the inhibitor at all due to pre-existing resistance mechanisms. A complete picture of the emergence of resistance would connect resistant subpopulations prior to treatment with their immediate drug response (over minutes to h), subsequent reactivation of kinase signaling and cell cycle progression (over h to days), and long-term transcriptional changes in these lineages (over weeks to months). This would enable the identification of key control points that could be exploited to improve treatment efficacy.

Our single-cell dose-titration assay, where cells are exposed to a sequence of several predetermined chemical stimuli, holds potential for exploring a wide range of cellular behaviors. We and others have demonstrated that perturbing cells reveal a much wider range of functional heterogeneity than observing cells at rest. In living tissues, cells are exposed to a variety of stimuli, and past exposure can amplify or negate responses to later stimuli based on updated internal cellular state^13,60^. Thus, we propose a new experimental paradigm, which we term “*cellular obstacle courses*”, where sequential stimuli are applied to cells and single-cell responses are tracked throughout the course of the experiment. Our dose-titration assay is a simple instance of a cellular obstacle course, which we use to infer dose responses in single cells. However, this approach has been used with more varied sequential stimuli including activation and inhibition of receptors and components in the ERK pathway^61^. Using this approach, the authors were able to dissect various contributions of receptors, pathway inhibition, and oncogenic mutations on ERK signaling. We envision that these experiments could be rationally designed, as we have demonstrated here, or could be employed as a high throughput screening tool, where multiple perturbations are combined in various orders and doses to map connections between cellular history and response to a particular perturbation^62^. The design space for such experiments is massive, and we have barely scratched the surface of potential applications of cellular obstacle courses.

Dose response assays are ubiquitous at all stages of pre-clinical drug development, from lead compound identification to clinical trial dose determination. Our computational model demonstrates that these assays ignore heterogeneity in cellular responses, and our experimental results validate that conclusion. Our results suggest four key considerations for drug development. First, our assay was performed in a 96-well plate and only requires readily available fluorescence imaging systems. Thus, it could be adapted to early-stage target identification, and previous efforts have already utilized biosensors for high-throughput characterization of interactions between drugs and signaling dynamics^63^. Secondly, previous drug-discovery paradigms have focused on achieving high levels of inhibition with limited success. It is currently unclear how specific levels of inhibition in single cells impact functional outcomes such as survival or migration. Our work supports a model where quantitative dose-signal-behavior relationships are measured and used to determine the optimal level of kinase inhibition required to achieve a specific therapeutic goal. Third, our work provides further context to dose selection in clinical trials and clinical medicine. Previous work has defined the therapeutic index of a drug as the ratio between the toxic and effective doses of a drug. In oncology a population-scale metric, such as reductions in tumor volume, typically define drug efficacy. Here, we demonstrate that the dose required for effectiveness against all cancer cells is likely to be increased by cellular heterogeneity, which will reduce the therapeutic index. Finally, our work reveals cell-line-dependent differences in the kinetics of kinase inhibition. The time required to achieve peak inhibition is a relatively understudied aspect of kinase inhibition and could help contextualize drug pharmacokinetics and pharmacodynamics. For instance, a drug with shorter peak plasma times but longer time to peak inhibition could yield more resistance because cells are not exposed to adequate drug concentrations for the required time. Thus, heterogeneity in cellular responses to inhibitors of kinases and other drug targets is crucial to understand across early stages of drug development.

Our work has several limitations. We utilize a kinase translocation reporter here and exploit the rapid kinetics of PI3K inhibition and Akt deactivation to measure single-cell dose responses. Akt signaling activity is commonly used as a measure of PI3K activation or inhibition, but dynamic imaging reporters may not exist for many other drug targets. Furthermore, our method may not be appropriate for drugs with different targets or mechanisms of action. Our measurements are also likely affected by intrinsic differences present in the cell population, including differences in cell cycle timing^64^. These differences may complicate interpretation of our result, but cancer cells in real tumors also exhibit differences in cell-cycle timing along with myriad other differences. Thus, studying these differences in vitro may provide vital information for accurately predicting how drugs behave in vivo. Effective cytotoxic drugs cause cell death, a binary endpoint without gradations needed to reproduce the dose-titration curves used for inhibition of kinases. Finally, our own results reveal that cells have different kinase deactivation kinetics, as measured by our KTRs. Thus, our assay may only be practical for cells and readouts that exhibit fairly rapid (on the order of minutes) deactivation kinetics. Nonetheless, our assay is compatible with a wide range of clinically relevant drugs, and novel reporters are continuously emerging, including reporters for metabolic activity^65,66^ and reporters localized to specific subcellular regions^67,68^. Future work will focus on adapting our approach to a broader range of cell lines and single-cell outputs.

## Methods

### Modeling single-cell dose responses

To model single-cell dose responses to kinase inhibitors, we first assumed that previous dose response experiments using western blots for phosphorylated and unphosphorylated proteins would function by averaging the amount of protein in each cell after cell lysis. Thus, if we simulated a dose response of active protein in individual cells, the population dose response would be represented by the average of all cells in the simulation at a given dose. We further assumed that the response of individual cells to drug could be described by a sigmoidal curve.

To simulate single-cell dose responses, we first generated distributions of dose response parameters for the traditional sigmoidal dose response equation (Eq. 1). The distributions used for each population in Fig. 1 are shown in Table 1. We centered the distributions around the measured population dose response for omipalisib applied to MCF-7 cells^37^, where the E_0_ was normalized to 1. For each simulated population, we sampled 1000 total cells. In the bimodal population simulation, we sampled 500 cells from each population, while in the resistant subpopulation simulation we sampled 950 cells from population 1 and 50 cells from population 2. For each cell, we sampled each parameter distribution independently to acquire a set of single-cell dose response parameters. We then used those dose response parameters to determine the dose response R(d) using Eq. 1 over a dose range from 10^−11 ^nM to 10^−5 ^nM. To calculate the simulated population dose response for each hypothetical population, we averaged across all cells at each dose.

**Table 1:** Parameters used for single-cell dose response simulations

		Parameters			
		*E*_0_	*E*_max_	Log_10_(EC_50_)	Log_2_(HS)
Populations	Low Variance	μ: 1 σ: 0.1	μ: 0.16 σ: 0.1	μ: −7.72 σ: 0.1	μ: −1.02 σ: 0.1
	High Variance	μ: 1 σ: 0.1	μ: 0.16 σ: 0.1	μ: −7.72 σ: 0.5	μ: −1.02 σ: 0.5
	Bimodal – population 1 (50%)	μ: 1 σ: 0.1	μ: 0.16 σ: 0.1	μ: −6.72 σ: 0.1	μ: −1.42 σ: 0.1
	Bimodal – population 2 (50%)	μ: 1 σ: 0.1	μ: 0.16 σ: 0.1	μ: −8.72 σ: 0.1	μ: −0.62 σ: 0.1
	Resistant Subpopulation – population 1 (95%)	μ: 1 σ: 0.1	μ: 0.16 σ: 0.1	μ: −7.72 σ: 0.1	μ: −1.02 σ: 0.1
	Resistant Subpopulation – population 2 (5%)	μ: 1 σ: 0.1	μ: 0.46 σ: 0.1	μ: −4.72 σ: 0.1	μ: −1.02 σ: 0.1

### Calculating dose response curves and surfaces

We acquire single-cell dose response surfaces (Figs. 2 and 6), we set a threshold that was a variable fraction of each cell’s baseline signaling. For Fig. 2b, we used a constant threshold of 60%. We then calculated how many cells at each dose were below their individual threshold. For Fig. 2c, we varied the threshold between 20% of basal signaling and 90% of basal signaling.

### Cell culture

We originally obtained MCF-7 cells from the ATCC (Manassas, VA, USA). We cultured MCF-7 cells in DMEM (Thermofisher, Waltham, MA USA), 10% fetal bovine serum, 1% glutamax, 1% penicillin/streptomycin, and plasmocin prophylactic (Invivogen, Toulouse, France) maintained at 37 °C and 5% CO_2_ in a humidified incubator. We trypsinized and re-suspended cells every 3 days to passage them. Short tandem repeat analysis was used to authenticate cell lines and we tested for mycoplasma when cells were initially passaged.

We obtained Vari-068 cells as a gift from the Sofia Merajver lab. We cultured these cells as described previously^13^.

### Stable expression of kinase translocation reporters

We stably expressed a nuclear histone 2B (H2B) marker and KTRs for Akt and ERK as described previously^13,69^. We used a PiggyBac transposon (Systems Biosciences, Palo Alto, CA, USA) with a vector containing H2B-mCherry, Akt KTR-mAquamarine, ERK KTR-mCitrine, and a puromycin selection marker, which we refer to as the pHAEP vector. After transposing, we selected positive cells by culturing cells using 5 μg/ml puromycin (ThermoFisher).

### Live-cell microscopy and perturbation experiments

For imaging experiments, MCF-7 pHAEP cells were plated at 5000 cells/well in 100 μl of imaging media in glass bottom 96-well plates (Cellvis, Mountain View, CA, USA). Imaging media consisted of 10% fetal bovine serum, 1% glutamax, 1% pen–strep, 1% sodium pyruvate, and 10 nM β-estradiol (Sigma-Aldrich, Millipore Sigma, St. Louis, MO, USA) in phenol-red free Fluorobrite DMEM (Thermofisher). We used an EVOS M7000 microscope with an on-stage incubator to perform live cell microscopy. The on-stage incubator was equilibrated at 37 °C and 5% CO_2_, and >80% humidity prior to adding the 96-well plate. 24 h after seeding, we added the cells to the microscope incubator. For imaging, we configured microscope settings to minimize light exposure. We used 10x magnification. We captured images of cells approximately every 3 minutes. We captured multiple contiguous fields within each well in an experiment and used autofocus on the H2B-mCherry channel on one field within each well at each timepoint. For dose response experiments, we imaged a pre-drug state for 20 minutes. Then, we added the lowest dose of alpelisib or omipalisib (SelleckChem, Houston TX, USA) and imaged for 1 h. We then added the next dose and imaged for 1 h, repeating this procedure until we added the highest drug concentration. Final drug concentrations were 0 nM, 0.15 nM, 0.6 nM, 2.4 nM, 9.8 nM, 39 nM, 156 nM, 625 nM, 2.5 μM, 10 μM, and 40 μM.

### Automated image processing

After acquiring images, we extracted single-cell signaling time tracks using an automated image processing algorithm as described previously^12,13,49^. First, adaptive thresholding is applied to nuclear images to identify nuclear pixels. We then dilate the nuclear mask by 5 pixels, while preserving the number of objects in the image. Dilating the nuclear mask identifies cytoplasmic pixels immediately outside of the nucleus. We preserve the object number so that pixels between two nuclei are only assigned to their nearest nuclei. The mean intensity in each nucleus and cytoplasm is calculated, which is used to calculate the cytoplasmic to nuclear ratio. Cells are connected through time by connecting nuclei with the most overlap in successive images. For dose response analysis, only cells that were tracked for the duration of the experiment were included.

### Single-cell dose response analysis

We identified dose responses in individual cells by averaging their measured log_2_(CNR) values over the timepoints from 50 to 60 min after each drug dose was applied. We performed the same procedure for both ERK and AKT KTRs. We calculated an untreated condition as the average of Akt or ERK at each timepoint between 10 min prior to the addition of the first drug dose. The procedure identified Akt and ERK signaling activity at 11 doses in hundreds of cells. To fit single-cell dose response curves, we used the lsqcurvefit() function in MATLAB R2022b (MathWorks, Waltham, MA USA). During fitting, we constrained the *E*_max_ and *E*_0_ values between −3 log_2_(CNR) and 3 log_2_(CNR), the EC_50_ between 0 and 40 μM, and the hill slope between 0 and 100. The experimental threshold inhibition surfaces (Fig. 6) were determined by first fitting dose response parameters to each cell in the experimental population and then repeating the same procedure as the simulated populations.

### Reporting summary

Further information on research design is available in the Nature Research Reporting Summary linked to this article.

### Supplementary information

Supplementary Material

Reporting summary

## Data Availability

Source data (raw images) and single-cell signaling data are available upon reasonable request.

## Code availability

Image processing code is available upon reasonable request. Dose response simulation code is available at https://zenodo.org/records/10892758.
