# Supplementary material for: Characterizing heterogeneous single-cell dose responses computationally and experimentally using threshold inhibition surfaces and dose-titration assays
Source: NPJ Syst Biol Appl. 2024 Apr 18;10:42. doi: 10.1038/s41540-024-00369-x (PMC11026493; doi:10.1038/s41540-024-00369-x)
Supplement: Supplementary file 1 — Supplementary Material [file 41540_2024_369_MOESM1_ESM.pdf]

Supplementary Information

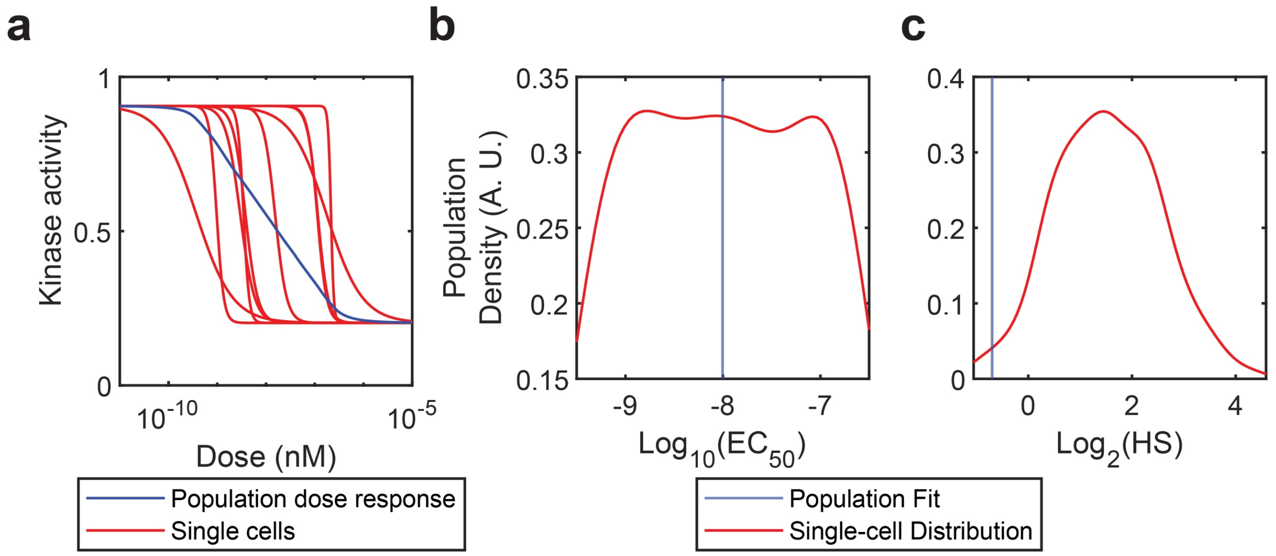

Supplementary Figure 1: Population level dose responses do not represent single cell behaviors. (a) Single cell dose responses (red) and averaged population dose response (blue) sampled from a population of cells with high HS and a range of EC_{50}s. (b-c) Comparison of inferred parameters from population average dose response (blue) and underlying distribution (red) for EC_{50} (b) and HS (c).

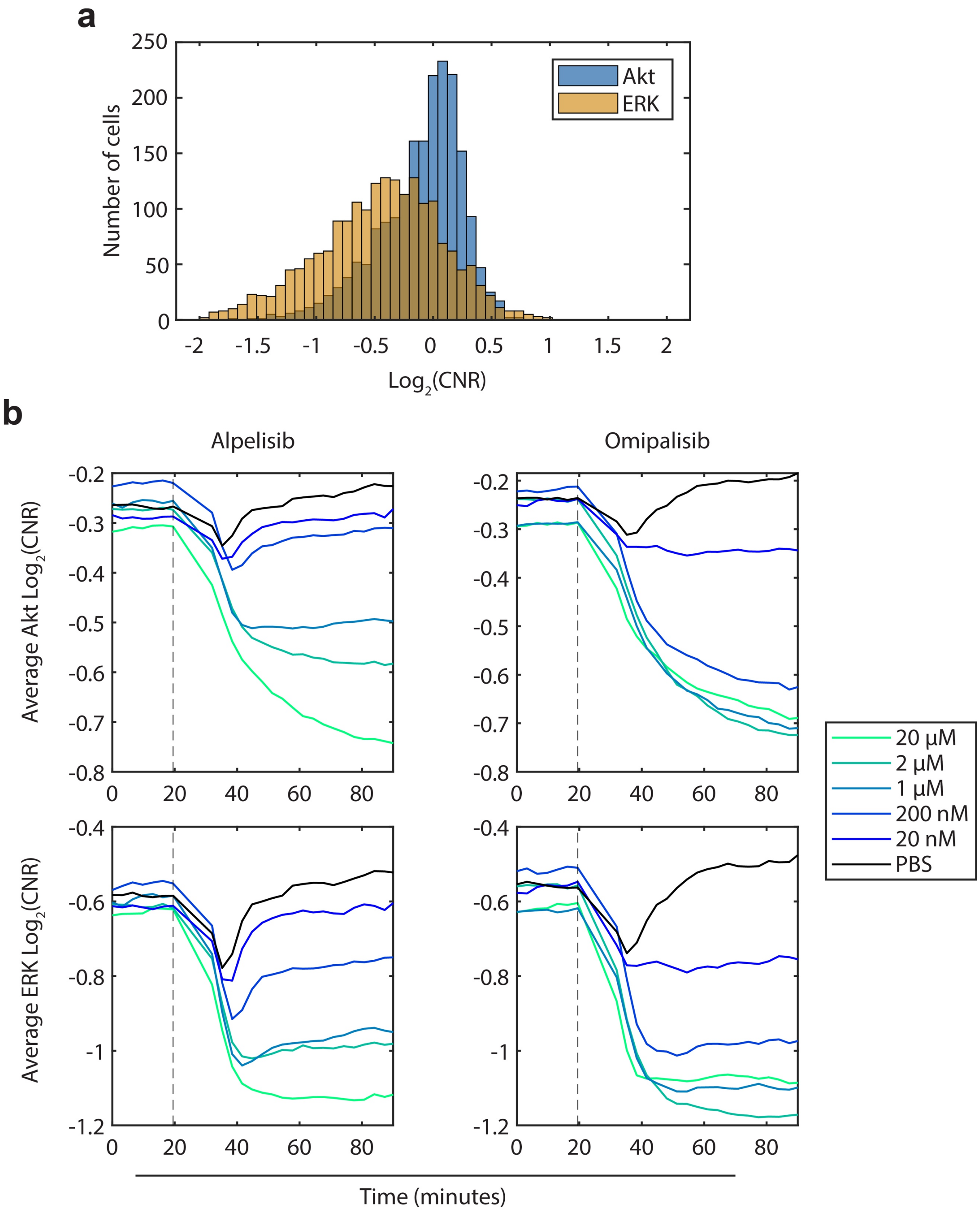

Supplementary Figure 2: Measuring Akt and ERK kinase activities in response to PI3K inhibition. (a) Histogram of Akt (blue) and ERK (yellow) $\log_2$(CNR) values for MCF-7 cells stably expressing Akt and ERK KTRs. N = 1951 cells from 3 wells. We quantify activities of each kinase based on the $\log_2$ ratio of cytoplasmic to nuclear fluorescence intensities ($\log_2$ CNR) for each reporter in a cell. A higher $\log_2$ CNR denotes greater kinase activity. (b) Average $\log_2$(CNR) for Akt (top) and ERK (bottom) in MCF-7 KTR cells exposed to alpelisib (left) or omipalisib (right). In each experiment, cells were exposed to the indicated concentration of PI3Ki or vehicle (PBS) at t = 20 minutes. The average over all cells tracked in the experiment is plotted. For Alpelisib, N is between 270 and 465 cells. For omipalisib, N is between 146 and 403 cells.

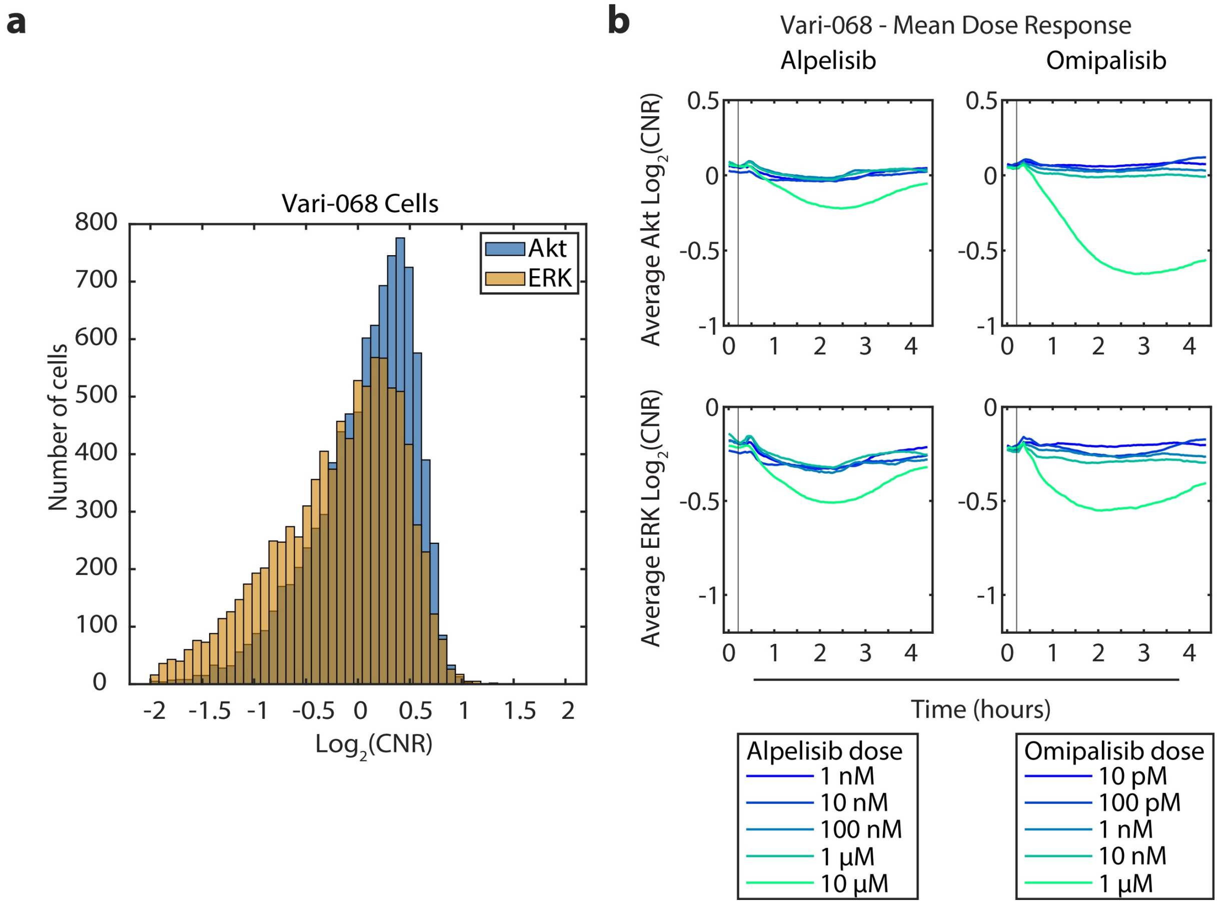

Supplementary Figure 3: Measuring Akt and ERK in responses to PI3K inhibition in Vari-068 cells. (a) Histogram of Akt (blue) and ERK (yellow) $\log_2$(CNR) values for MCF-7 cells stably expressing Akt and ERK KTRs. N = 9219 cells from 6 wells. (b) Average $\log_2$(CNR) for Akt (top) and ERK (bottom) in Vari-068 KTR cells exposed to alpelisib (left) or omipalisib (right). Doses are given in legend below figure. Note different doses used for each drug. In each experiment, cells were exposed to the indicated concentration of PI3Ki at t = 20 minutes. The average over all cells tracked in the experiment is plotted. For Alpelisib, N is between 592 and 1035 cells. For omipalisib, N is between 631 and 1248 cells.

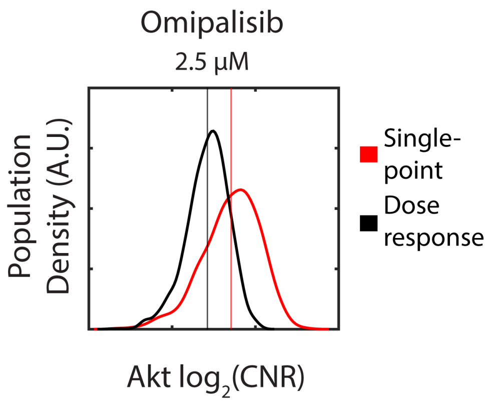

Supplementary Figure 4: Comparing dose-response and single-point inhibition in Vari-068 cells. Comparison of Akt signaling distribution when exposed to 2.5 μM of omipalisib in a single point experiment (red) and a dose-response experiment (black). Vertical lines indicate the population means of the distributions. For comparison with figure 1c. Lower doses of omipalisib and alpelisib treatment are omitted because they do not inhibit Akt in Vari-068 cells.

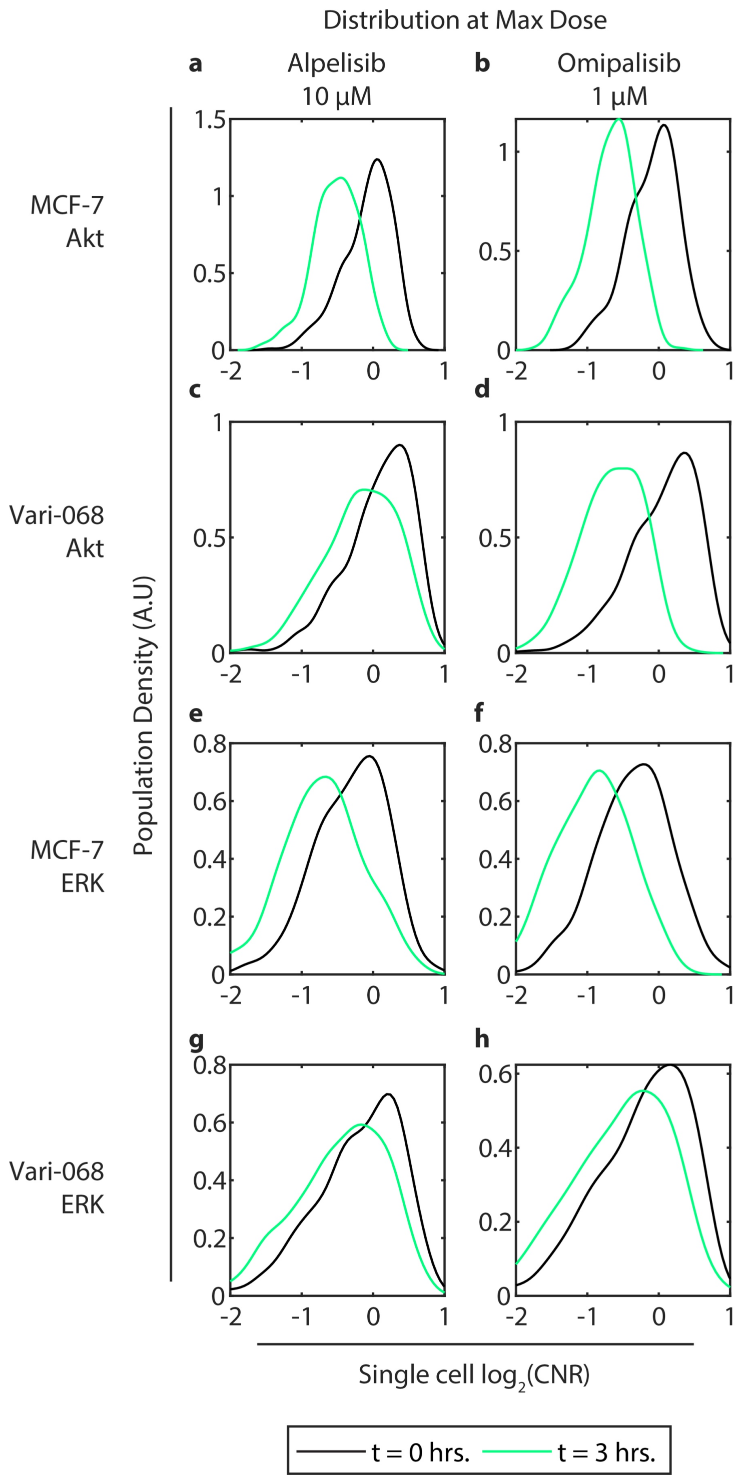

Supplementary Figure 5. Heterogeneity in signaling at maximal inhibition. Distributions of signaling activity before inhibition (black) and at maximal inhibition (green, t = 3 hrs., maximum dose from supplemental figures 1 and 2). (a-d) Akt signaling activity for MCF-7 (a,b) and Vari-068 (c, d) cells exposed to 10 µM alpelisib (a, c) or 1 µM omipalisib (b, d). e-h: Same as a-d for ERK signaling activity.
